# Supplementary material for: Sustainable Antibacterial and Antiviral High-Performance Copper-Coated Filter Produced via Ion Beam Treatment
Source: Polymers (Basel). 2022 Mar 2;14(5):1007. doi: 10.3390/polym14051007 (PMC8914895; doi:10.3390/polym14051007)
Supplement: Supplementary file 1 [file polymers-14-01007-s001.zip › polymers-1600226-supplementary.pdf]

Supplementary Materials

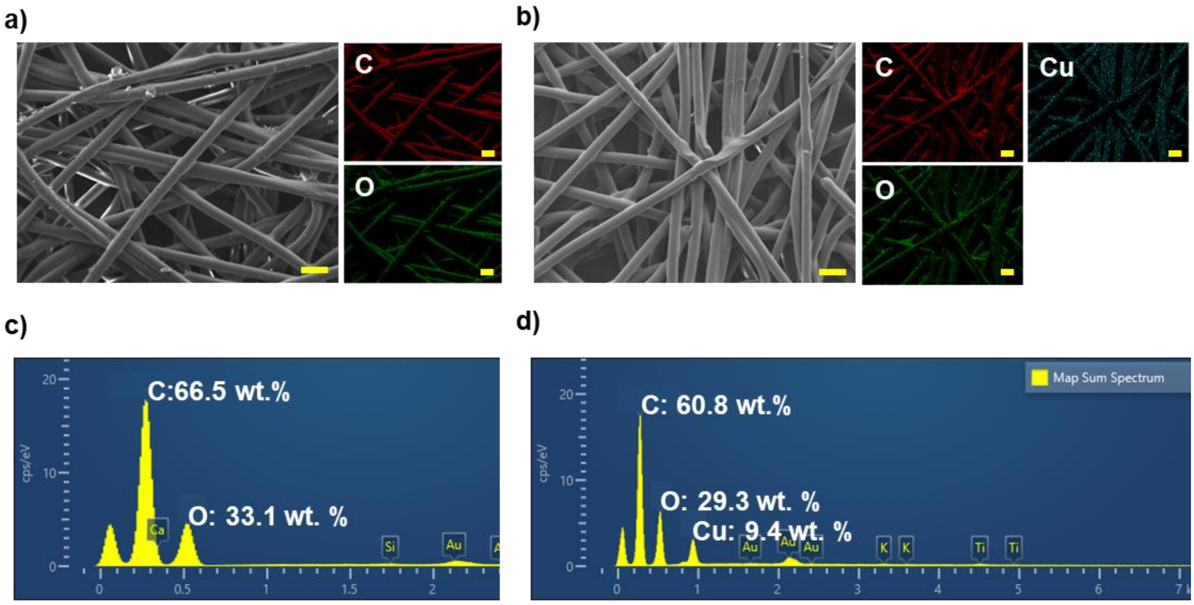

Figure S1. SEM (grayscale) and EDS mapping (color) images (scale bar: 100 µm) of the surfaces of the a) bare filter and b) copper-coated filter after ion beam treatment by Ion Beam 2. Atomic weight percentages of the surfaces of the c) bare filter and d) copper-coated filter after treatment by Ion Beam 2.

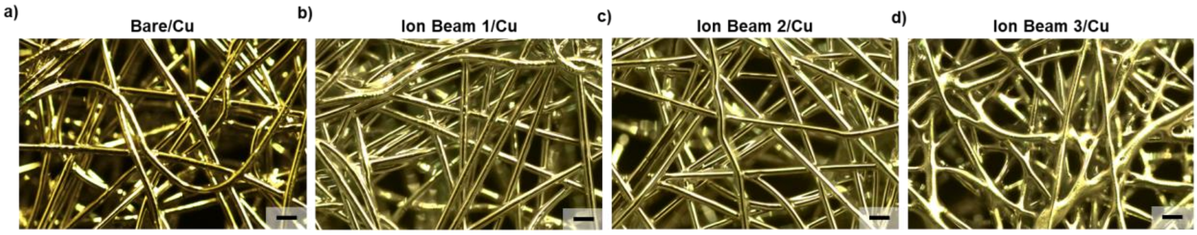

Figure S2. : Optical microscope images (obtained under ultraviolet light with a peak wavelength of 360 nm; scale bar: 100 μm) of copper-coated filters treated with a) no ion beam (bare filter), b) Ion Beam 1, c) Ion Beam 2, and d) Ion Beam 3.

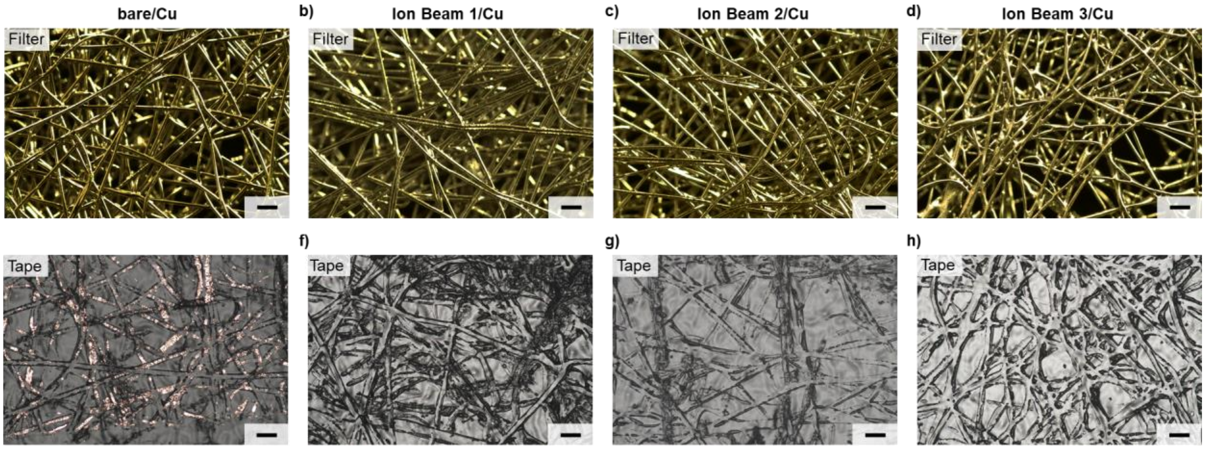

Figure S3. Optical microscope images (obtained under ultraviolet light with a peak wavelength of 360 nm; scale bar: 100 μm) of copper-coated filters after the peeling test and treated with a) no ion beam (bare filter), b) Ion Beam 1, c) Ion Beam 2, and d) Ion Beam 3. Optical microscope images (obtained under white light; scale bar: 100 μm) of the tape surfaces after the peeling test for e) no ion beam (bare filter), f) Ion Beam 1, g) Ion Beam 2, and h) Ion Beam 3.

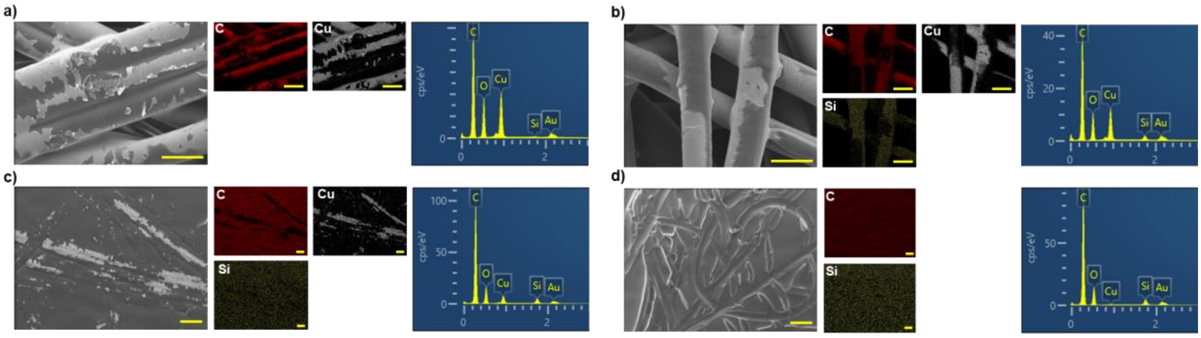

Figure S4. SEM and EDS mapping images (scale bar: 50 μm) of copper-coated filter surface after tap e peeling test of a) bare and b) Ion Beam 1. SEM and EDS mapping images (scale bar: 100 μm) of tape surface after tape peeling test of a) bare and b) Ion Beam 1.

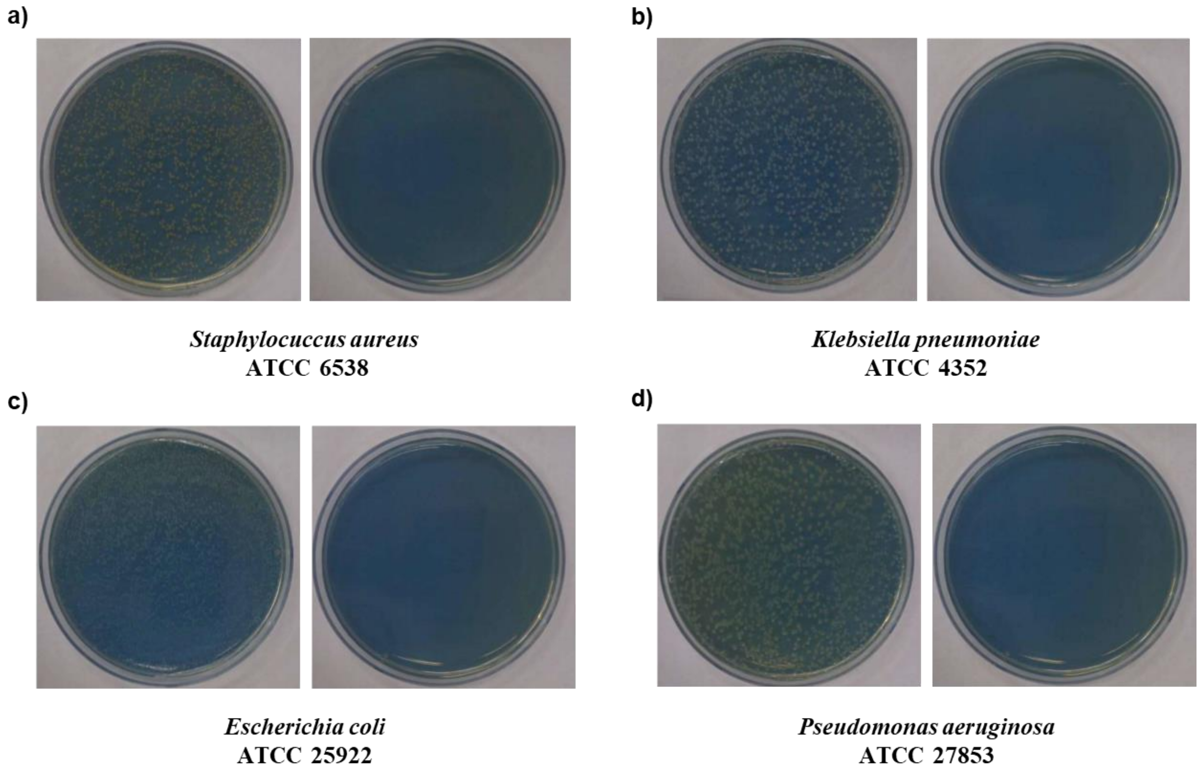

Figure S5. Antibacterial test results for a) Staphylocuccus aureus ATCC 6538, b) Klebsiella pneumoniae ATCC 4352, c) Escherichia coli ATCC 25922, and d) Pseudomonas aeruginosa ATCC 27853. In each panel, the left col umn shows the control group exposed to the bare filter, and the right column shows the experimental group expo sed to the copper-coated filter.

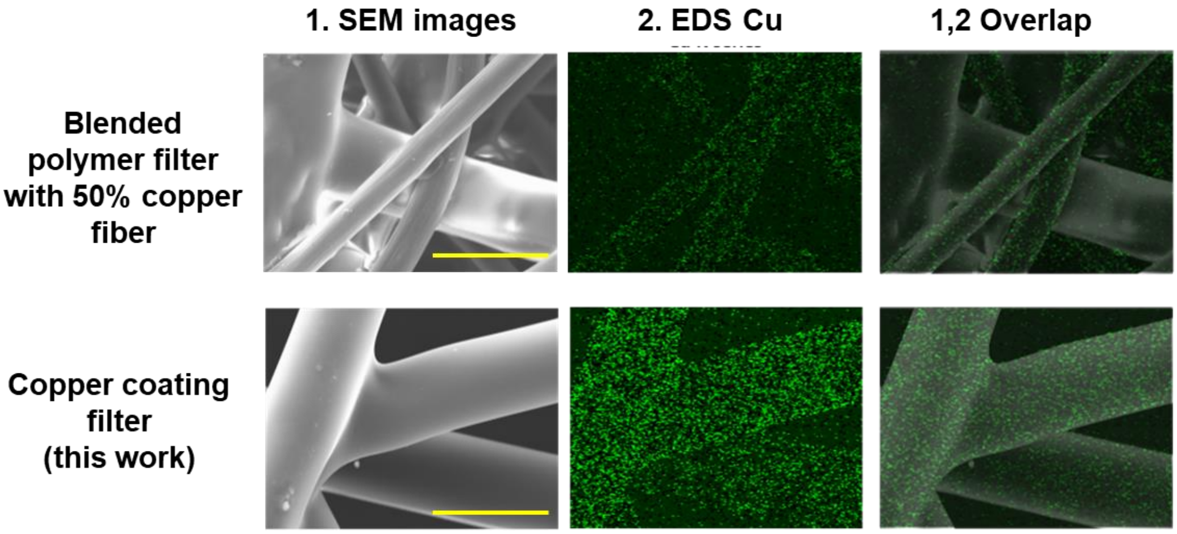

Figure S6. Differences in copper distribution between commercially available 50% copper fiber–blended polymer filters and copper-coated filters (this work). The copper exists only on thin fibers in the blen ded polymer filter. However, it is distributed evenly in the copper-coated filter.

Table S1. Vacuum ion-beam surface treatment and sputtering process conditions.

Step 1	Ion Beam 1	Ion Beam 2	Ion Beam 3
Current (mA)	50	100	100
Applied voltage (kV)	0.6	1	2
Stage moving speed (meter/minute)	0.1	0.1	0.1
Number of moving	2	2	2

Step 2	Cu Sputtering
Gas / Flow rate (sccm)	Ar / 40
Applied power (W)	200 W
Stage moving speed (meter/minute)	0.6
Number of moving	4
